# High royal jelly production does not impact the gut microbiome of honey bees

**DOI:** 10.1186/s42523-021-00124-1

**Published:** 2021-09-13

**Authors:** Megan E. Damico, Olav Rueppell, Zack Shaffer, Bin Han, Kasie Raymann

**Affiliations:** 1 Biology Department, University of North Carolina at Greensboro, Greensboro, NC 27412 USA; 2 Department of Biological Sciences, University of Alberta, Edmonton, AB T6G 2E9 Canada; 3 Institute of Apicultural Research, Chinese Academy of Agricultural Science, Beijing, 100093 China; 4 Present Address: School of Medicine, Wayne State University, Detroit, MI 48201 USA

## Abstract

**Background:**

Honey bees are not only essential for pollination services, but are also economically important as a source of hive products (e.g., honey, royal jelly, pollen, wax, and propolis) that are used as foods, cosmetics, and alternative medicines. Royal jelly is a popular honey bee product with multiple potential medicinal properties. To boost royal jelly production, a long-term genetic selection program of Italian honey bees (ITBs) in China has been performed, resulting in honey bee stocks (here referred to as RJBs) that produce an order of magnitude more royal jelly than ITBs. Although multiple studies have investigated the molecular basis of increased royal jelly yields, one factor that has not been considered is the role of honey bee-associated gut microbes.

**Results:**

Based on the behavioral, morphological, physiological, and neurological differences between RJBs and ITBs, we predicted that the gut microbiome composition of RJBs bees would differ from ITBs. To test this hypothesis, we investigated the bacterial composition of RJB and ITB workers from an urban location and RJBs from a rural location in China. Based on 16S rRNA gene profiling, we did not find any evidence that RJBs possess a unique bacterial gut community when compared to ITBs. However, we observed differences between honey bees from the urban versus rural sites.

**Conclusions:**

Our results suggest that the environmental factors rather than stock differences are more important in shaping the bacterial composition in honey bee guts. Further studies are needed to investigate if the observed differences in relative abundance of taxa between the urban and rural bees correspond to distinct functional capabilities that impact honey bee health. Because the lifestyle, diet, and other environmental variables are different in rural and urban areas, controlled studies are needed to determine which of these factors are responsible for the observed differences in gut bacterial composition between urban and rural honeybees.

**Supplementary Information:**

The online version contains supplementary material available at 10.1186/s42523-021-00124-1.

## Introduction

Honey bees (*Apis mellifera*) are used for pollination services for crops as well as for honey production across the world. In addition to honey, other bee products such as pollen, propolis, royal jelly and wax are also used for food, cosmetics products, and as alternative medicines [1, 2]. In particular royal jelly (RJ) is the bee product that is believed to be the most promising for treating human diseases and illnesses [1] and because of the perceived health benefits and antibacterial properties, the demand for RJ is high [2]. Royal jelly is a nutrient-rich substance that is secreted by the hypopharyngeal and mandibular glands of honey bee workers. It is the sole food source for honey bee queens for their entire life and it is also fed to larvae during their first few days of development [3]. Decades of genetic selection of Italian honey bees (ITBs) in China resulted in a stock of royal jelly producing bees (RJBs) that produce 10 times more RJ than ITBs and are genetically distinct [4]. Now, RJBs are the largest commercial producers of RJ in the world, producing over 90% of the total RJ on the market which annually grosses over 2.5 billion dollars [1].

Many factors are correlated with the higher RJ production phenotype of the RJBs [5]. The acini of hypopharyngeal glands (HGs) are significantly larger in RJBs than ITBs and have the potential to secrete RJ earlier [6]. Upregulated pathways of protein synthesis and energy metabolism have been identified to support HGs performance in RJBs for the elevated RJ production [7]. In addition, enhanced lipid synthesis and transport pathways in mandibular glands of RJBs may contribute to higher RJ production [8]. Furthermore, 4-day old RJB larvae possess an elevated number of proteins compared to unselected ITBs in their hemolymph that are involved in amino acid and protein synthesis [9]. In recent years, neurobiological correlates of the RJB “syndrome” have been identified, such as a stronger olfactory response to brood pheromone that is presumably linked to up-regulated chemosensory proteins and antennal metabolism [10] and signal transduction and energy and nutrient metabolism pathways in the central nervous system [11, 12]. Moreover, RJBs have higher levels of neuropeptides implicated in regulating water homeostasis, brood pheromone recognition, foraging capacity, and pollen collection compared to ITBs [11]. Although continuous efforts have been devoted to elucidating the molecular basis of increased RJ yields, one factor that has not been considered is the role honey bee-associated microbes (the microbiome) might play in RJ production.

Honey bees acquire their gut microbiome mainly through social interactions with their sisters in the hive after emergence. The bacteria that reside in the honey bee gut are specific to corbiculate bees and the microbiome composition is highly conserved across individual honey bees [13]. The honey bee gut microbiome consists of nine bacterial taxa that make up ~ 95% of the entire gut community with the remaining 5% belonging to facultative members [14]. Five of these nine bacterial taxa represent the core microbiota and include: *Snodgrassella alvi, Gilliamella spp., Lactobacillus* Firm-5, *Lactobacillus* Firm-4, and *Bifidobacterium* spp. [14]. These five members are considered core because they are found in all healthy honey bees globally at relatively consistent proportions [13]. The core taxa are present in all honey bees, but differences among individuals and colonies can be seen in the relative frequencies of the major taxa, the amount of strain diversity within each of the core member, and the presence and abundance of environmental (transient) or pathogenic species [13]. The gut microbiome of honey bees has been shown to play an important role in bee nutrition, development, behavior, and immune response [15–17]. Additionally, differences in microbiome composition have been observed based on genotype, environmental landscape, geographical location, season, and diet [18–21]. Several studies in other insects have revealed that gut bacterial communities have neurological and behavioral effects on their host, including development, social interactions, cognition, and chemical communication [22]. Given the nutritional, behavioral, morphological, physiological, and neurological differences between RJBs and ITBs, we hypothesized that the gut bacterial composition would differ between these two stocks of honey bees and if true, could indicate that bacteria have an impact on RJ production or vice versa.

Here we investigated the gut bacterial composition of RJBs and ITBs from an urban location and RJBs from a rural location in China. We predicted that RJB and ITBs would differ in bacterial composition, regardless of environment, due to the numerous biological differences between these two stocks of honey bees. However, based on 16S rDNA profiling, we did not find any evidence that RJBs harbor a unique bacterial microbiome when compared to ITBs. Instead, we observed differences between honey bees located in urban versus rural environments.

## Results

**Fig. 1:**
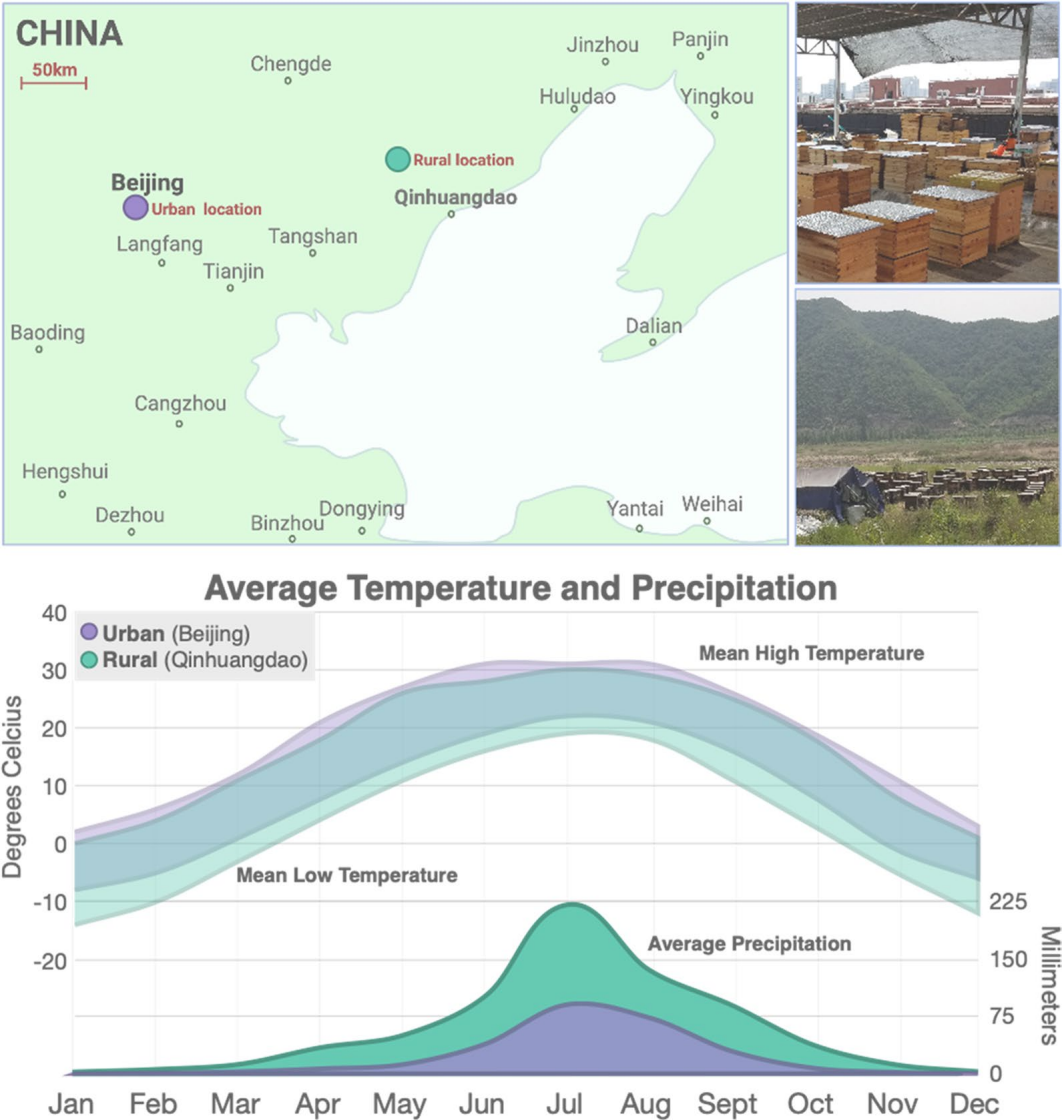
Sampling locations of ITBs and RJBs from China. Honey bees were sampled from six RJB and six ITB hives located on the rooftop of the Chinese Academy of Agricultural Sciences in Beijing, China (i.e. urban, pictured in top right) and from six RJB hives located in Zhuzhangzi township, Qinglong County, Qinhuangdao, Hebei province, China (i.e. rural, pictured in bottom left). The mean monthly temperature high and low and mean amount of precipitation for each location are shown in the bottom panel. The rural location is cooler in terms of average monthly temperature high and low and also receives higher precipitation, particularly during the summer when bees were sampled (June 2018). The graph was created based on climate data from https://www.timeanddate.com

**Fig. 2:**
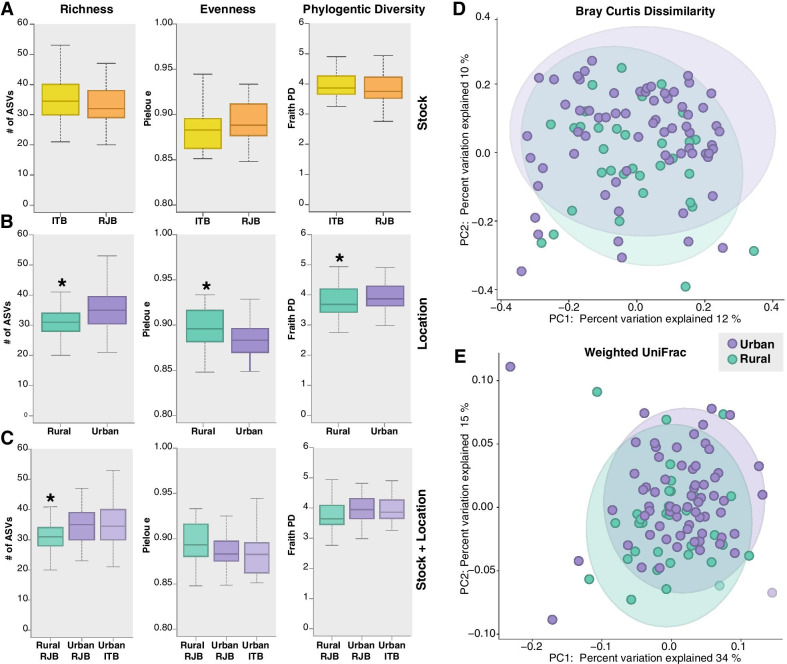
Alpha and beta diversity analyses. Alpha diversity comparisons of the gut microbiomes of **a** ITBs and RJBs, **b** rural and urban bees, and **c** urban ITB and RJBs and rural RJBs based on richness (# of ASVs), evenness (Pielou's evenness index) and phylogenetic diversity (Faith’s phylogenetic diversity index). * = *p*-value < 0.05, Kruskal Wallis test. Beta diversity comparisons of the gut microbiomes of urban and rural bees. Principal coordinate analysis plots based on **d** Bray Curtis dissimilarity and **e** weighted UniFrac. Significance was tested using PERMANOVA with 999 permutations (Bray Curtis, *p* = 0.014; weighted UniFrac, *p* = 0.015). Ellipses represent the 95% confidence interval

We sampled RJBs from urban and rural China and ITBs from urban China, (Fig. 1) and performed 16S rRNA gene profiling of the gut bacterial communities. Comparison of bacterial diversity within individuals (alpha (α) diversity) did not reveal any significant differences between stocks (i.e. RJBs and ITBs). However, differences in α-diversity were observed between the gut microbiomes of honey bees from urban versus rural environments, regardless of stock (Fig. 2a). Specifically, gut bacterial communities of honey bees from the urban apiary displayed higher ASV (amplicon sequence variant) richness, were less even, and had higher phylogenetic diversity than bees from the rural apiary (Fig. 2b). When comparing α-diversity among experimental groups, the rural RJBs possessed a significantly lower number of ASVs than both the urban RJBs and ITBs (Fig. 2c), consistent with the results observed when comparing rural versus urban samples overall. However, no significant differences were observed in terms of evenness and phylogenetic diversity between rural RJBs and urban RJBs or ITBs (Fig. 2c).

When considering beta (β) diversity, or bacterial divergence between groups, no significant differences were observed between ITB and RJB stocks (Additional file 1: Figure S1: Bray Curtis dissimilarity, *p* = 0.083; weighted UniFrac, *p* = 0.19). However, urban and rural honey bees were found to be significantly different from each other (Fig. 2d–e; Bray Curtis, *p* = 0.014; weighted UniFrac, *p* = 0.015). Furthermore, at the group level, rural RJBs were significantly different from urban ITBs based on both metrics (Additional file 1 Figure S1; Bray Curtis, *p* = 0.016 and weighted UniFrac, *p* = 0.037). Rural RJBs were found to be significantly different than urban RJBs based on weighted unifrac (*p* = 0.016) but not Bray Curtis (*p* = 0.167) (Additional file 1: Figure S1).

**Fig. 3:**
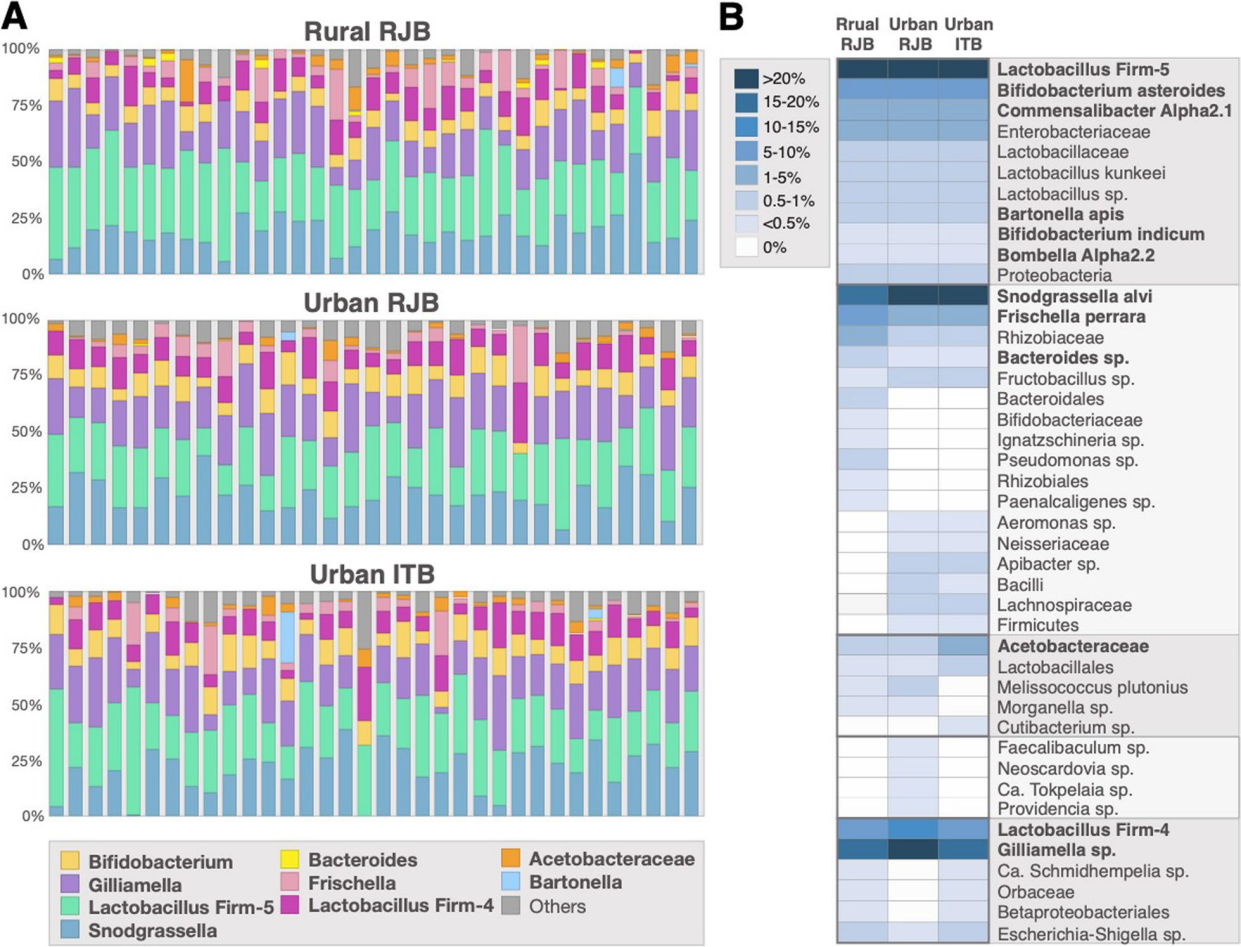
Relative abundance of bacteria in the gut microbiomes of urban ITB and RJBs and rural RJBs. **a** Relative abundance of the nine honey bee-associated taxa in each individual. **b** Heatmap of the average relative abundance of all taxa detected in rural RJBs, and urban RJBs and urban TBs clustered to the highest taxonomic level possible

Next, we analyzed taxonomic diversity in the samples. ASVs were clustered into 10 groups: the nine honey bee-associated taxa and “others” (Fig. 3a). All samples contained the five core honey bee gut microbiome taxonomic groups, *Lactobacillus* Firm4 and Firm5, *Bifidobacteria*, *Gilliamella*, and *Snodgrassella* (Fig. 3a). The five core taxa accounted for, on average, 86% of the total bacterial abundance in all bees analyzed. The other four frequently observed honey bee-associated taxa *Frischella*, *Bacteroides*, *Bartonella*, *Acetobacteraceae* were present in most of the bees analyzed and comprised, on average, 7% of the community (Fig. 3a, Additional file 2: Dataset S1). As reported in previous studies [13, 23, 24], these nine taxonomic groups made up over 93% of the total bacterial population in all honey bees analyzed. Thirty-one “other” taxa, most of which we were unable to classify to the genus level and are thought to be transient [13], were found in some bees at low relative abundance (Fig. 3a, Additional file 2: Dataset S1).

To visualize differential abundance of taxa, ASVs were clustered based on taxonomic assignment using the Bee Gut Microbiota-Database [25] database the average relative abundance of each taxon across groups was plotted using a heatmap (Fig. 3b). Taxa were classified to the highest level possible based on the database used as well as manual blast searches on the non-redundant nucleotide database on NCBI (https://blast.ncbi.nlm.nih.gov/). A few taxa were found to be uniquely present (*Melissococcus plutonius* and *Morganella* sp.) or absent (*Cutibacterium* sp.) in RJBs from both locations. Six taxa were only found in rural RJBs and six others were shared between urban RJBs and ITBs but completely absent in rural RJBs (Fig. 3b). Four taxa were unique to urban RJBs and three taxa were shared between urban ITBs and rural RJBs but absent in urban RJBs (Fig. 3b). Overall, based on presence/absence of taxa, more differences were observed between urban and rural bees than between stocks.

**Fig. 4:**
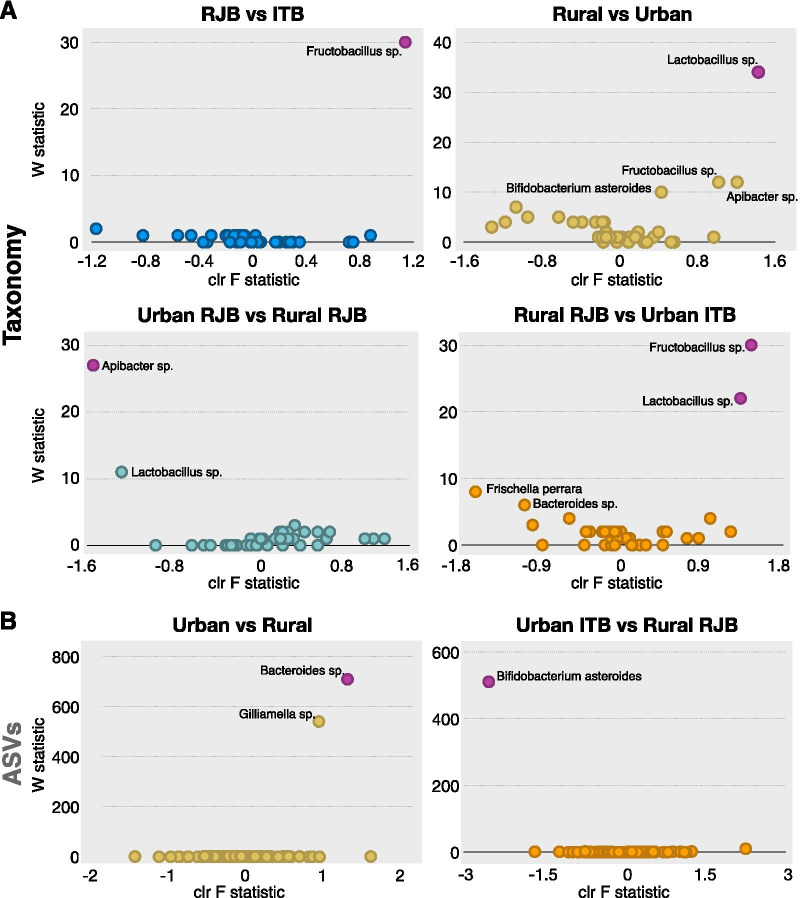
ANCOM differential abundance volcano plots. The y-axis represents the W statistic value or the number of times of the null-hypothesis was rejected for a given **A)** taxonomic cluster or **B)** ASV. The x-axis value represents the centered log ratio (clr) transformed F statistic (the effect size difference for a particular taxon/ASV between groups). Taxa/ASVs with reject null-hypothesis are shown in pink and labeled with taxon name, all other taxa are colored the same within a given plot. Taxa/ASVs that were not significant but displayed a higher than average W value are also labeled with taxon name

To determine if there were any significant compositional differences across groups, we used the statistical framework ANCOM [26]. When ASVs were clustered based on taxonomy, one taxon was found to be significantly more abundant in ITBs than in RJBs: *Fructobacillus* sp. (Fig. 4a). Comparison of rural versus urban bees revealed four taxa that were present in higher abundance (*Lactobacillus* sp., *Apibacter* sp. *Fructobacillus* sp. and *Bifidobacterium asteroides*) in urban bees, but only *Lactobacillus* sp. was statistically significant (Fig. 4a). Consistent with the overall urban versus rural comparison, urban RJBs had a higher abundance of *Lactobacillus* sp. and *Apibacter* sp. than rural RJBs, with only the latter being significant, and urban ITBs had a higher abundance of *Fructobacillus* sp. and *Lactobacillus* sp. than rural RJBs (both statistically significant). In addition, rural RJBs displayed a higher abundance of *Frishella perrara* and *Bacterioides* sp. than urban ITBs, although not significant. No statistically significant differences were found between urban RJBs and urban ITBs (Additional file 3: Figure S2).

At the individual ASV-level, two ASVs corresponding to *Gilliamella* sp. and *Bacteroides* sp. were found in higher relative abundance in rural bees when compared to urban bees but only the difference in *Gilliamella* sp. ASV was statistically significant (Fig. 4b). An ASV classified as *Bifidobacterium asteroides* was observed in higher abundance in urban ITBs than in rural RJBs (Fig. 4b). No significant differences in relative abundance were found at the ASV-level between RJBs and ITBs, urban RJBs and urban ITBs, or urban RJBs and rural RJBs (Additional file 3: Figure S2).

## Discussion

Overall, our findings did not support the hypothesis that RJBs and ITBs differ in bacterial composition or community structure. We did not find any differences between these two stocks based on α- or β-diversity analyses and only one taxon was found to be statistically different between RJBs and ITBs; ITBs possessed a higher abundance of *Fructobacillis* sp. than RJBs. *Fructobacillus* sp. is a nectar associated bacterium that is commonly found in low abundance in the honey bee gut microbiome [27]. The reason for the higher relative abundance of *Fructobacillus* sp. is likely attributed to the fact that rural RJBs had a very low average relative abundance of this taxon (0.01%), and urban RJBs and ITBs possessed a higher average relative abundance (0.3% each), so when rural and urban RJBs were combined it decreased the overall relative abundance of *Fructobacillus* sp. in RJBs compared to ITBs. Evidence for this hypothesis is provided by the statistically higher relative abundance of *Fructobacillus* sp. in urban ITBs than in rural RJBs (Fig. 4a). Two taxa were found only in RBJs, (*M. plutonius* and *Morganella* sp.) and one taxon was absent from RBJs but present in ITBs (*Cutibacterium* sp.), but all three of these taxa were only present in a few bees (1–6 total) and were found at low relative abundance (between 0.3 and 3.0%). *M. plutonius* is a honey bee brood pathogen and *Morganella* sp. and *Cutibacterium* sp. are not typically associated with honey bees. Thus, their presence in some RBJs is not likely to correspond to royal jelly production.

Although we found very little evidence for differences in gut bacterial composition between RJB and ITBs, we observed significant differences in the bacterial community structure between honey bees located in urban versus rural environments based on α- and β-diversity analyses and differential abundance. The gut bacterial communities of honey bees from the urban apiary possessed more ASVs, were less even, and had higher phylogenetic diversity than rural bees. Moreover, rural RJBs had a significantly lower number of ASVs when compared to both the urban RJBs and ITBs. We also found that the bacterial composition of urban honey bees was significantly different from rural honey bees, with both urban ITBs and RJBs being more similar to each other than to the rural RJBs. However, the major differences observed between urban and rural bees were due to variation in the relative abundance of non-core taxa, while the core taxa were relatively invariant. This is consistent with numerous previous studies that have demonstrated that all honey bees possess a highly conserved core gut microbiome [13, 14, 23, 28, 29].

Multiple studies have shown that the microbiome is important for metabolism, immune response, growth and development in honey bees [15–17, 28, 30–33]. The honey bee gut microbial community is not only highly conserved across individuals of *A. mellifera* but the members of the community have co-evolved with each other and their host for millions of years [13, 34]. This co-evolution has resulted in species specialization and syntrophy (or cross-feeding) between members in the community [13, 34]. The functional roles of each core bee gut member and their cross-feeding interactions have been relatively well-characterized [17, 29, 35–39]. However, since all the taxa found to be differentially abundant between urban and rural bees are not part of the core microbiome, it is unclear how these differences would impact honey bee health. One interesting taxon found to be unique in urban bees, *Apibacter* sp. is not commonly found in *A. mellifera* but is endemic to the Asian honey bee species, *Apis dorsata* and *Apis cerana* [40]. The urban apiary was in close proximity to some *A. cerana* colonies, indicating presumably a cross-species transfer of *Apibacter* sp. Whether their colonization of *A. mellifera* is only transient or signifies a more permanent host expansion remains to be determined. However, a recent study has shown that *Apibacter* can colonize the gut of *A. mellifera* under lab conditions, suggesting the potential of host expansion [41].

The causes of the differences between rural and urban honey bees are unclear, but they could be due differences in food, exposure to environmental bacteria, or other environmental variables. The two taxa sthat differed the most (*Fructobacillis* sp. *Lactobacillis* sp.) between urban and rural bees are associated with nectar and pollen but the main food plant of both our urban and rural locations in June was *Vitex negundo* var. heterophylla (Franch.) Rehder, commonly known as the Chinese chaste tree. Our rural bees were sampled on an overcast day (June 22, 2018), and the urban bees were sampled on a sunny day (June 23, 2018). One hypothesis is that bees from the rural apiary were not actively foraging during the sampling day due to overcast weather, so they were not actively exposed to nectar and pollen associated microbes on the day of sampling. Additionally, sampling on an overcast day when most foragers are present in the hive could also have resulted in sampling more foragers than would be sampled on a sunny day. Thus, the observed differences between urban and rural bees could also be due to differences in sampling, as nurses and foragers have been shown to display differences in bacterial composition [21]. Aside from the weather on the day of sampling, the average yearly temperatures in Beijing are higher than in Qinhuangdao and average precipitation, specifically from May–September, is lower in Beijing (Fig. 1). It is possible that the differences in climate could impact the honey bee microbiome. It has been shown that the bacterial community structure of winter honey bees differs from summer honey bees [21], but the impact of different yearly average temperatures and precipitation on the gut microbiome of honey bees has not been specifically investigated. Exposure to anthropogenic chemicals such as pesticides has been shown to cause changes in the abundance of bacterial taxa in the bee gut microbiome [42, 43]. The gut microbiomes of urban and rural honey bees could result from differential exposure to anthropogenic chemicals between the two groups but we lack exposure data to further assess this potential explanation. However, a similar hypothesis was put forth to explain the variation in microbiome composition observed between honey bees located in different proximities to agricultural fields treated with pesticides [17]. Furthermore, we lack data from our two sampling locations on the environmental abundance of potential colonizers that could alter the bacterial gut communities. There are also other factors that might explain the differences between our urban and rural bees, such as hive management and pathogen load, etc. that seem less likely but cannot be ruled out.

Although we predicted that RJBs and ITBs would differ in microbiome composition due their differences in behavior, physiology, and neurobiology that relate to nutrition, we did not find strong evidence for variation between the two stocks. We might have missed important functional differences because this study was limited to 16S amplicon sequencing: We were only able to profile the microbial communities at the ASV-level, which arguably does not accurately correspond to species [44]. Multiple recent studies have demonstrated that extensive strain-level diversity exists within each of the core honey bee gut microbiome taxa [17, 29, 35, 37, 38, 40, 44–48] and although the core taxa are conserved across honey bees, individuals (even from the same hive) can possess very different strains [46, 47]. Additionally, it has been shown that different strains of the same species can possess very distinct functional capabilities, e.g., metabolic capabilities and variation in tolerance or resistance to chemicals [17, 36–38, 43, 46]. Because 16S amplicon sequencing does not detect strain-level diversity or provide information about functional capabilities, we were unable to fully investigate whether the microbial communities of RJBs and ITBs differ in function. Thus, we cannot exclude that there are strain-level differences between these stocks that correspond to functional differences relating to the production of royal jelly. In order to investigate this hypothesis further, a large-scale metagenomic study would have to be conducted. Moreover, the hypopharyngeal glands possess a microbiome that is distinct from the gut microbiome [49] and may play a more direct role in RJ production. However, it remains to be evaluated whether the hypopharyngeal gland microbiome of RJBs is different from ITBs.

## Conclusions

Overall, our results suggest that the environment rather than stock is selecting for a specific microbiome composition in honey bees. Further studies are needed to investigate if the observed differences in relative abundance of taxa between the urban and rural bees represent a general pattern and correspond to distinct functional capabilities that impact honey bee health. Because the lifestyle, diet, and a number of other environmental variables are different in rural and urban areas, controlled studies are needed to determine which of these variables play a role in shaping the gut microbiome composition in honeybees.

## Methods

### Sample collection

Honey bees were sampled from six RJB and six ITB hives located on the rooftop of the Chinese Academy of Agricultural Sciences in Beijing, China (i.e., urban) and from six RJB hives located in Zhuzhangzi township, Qinglong County, Qinhuangdao, Hebei province, China (i.e., rural). The rural bees were collected on June 22, 2018 (overcast day with temperature high of 32 °C and low of 16 °C). and the urban samples were collected on June 23, 2018 (sunny day with temperature high of 36 °C and low of 23 °C). Differences in royal jelly production among our three populations was not precisely quantified, but visual inspection confirmed the previously reported differences between royal jelly bees and unselected stock [10]. None of the hives from either location had a recent history of chemical therapeutic or preventative treatments (e.g., antibiotics, fungicides or miticides). For each hive, eight honey bees were randomly sampled from a brood frame (thus were likely nurse bees) resulting in 48 bees from each stock and location (144 bees total). Bees were immobilized at 4 °C and the entire gut (i.e., stomach, ileum, and rectum) of each bee was extracted in the lab using sterile forceps and immediately placed in 500 μl of RNAlater™ (Fisher Scientific). Samples were stored at − 80 °C and then transported on dry ice to Greensboro (North Carolina, USA) for processing and analysis. Ideally, we would also have sampled ITBs from rural China, but this genotype was not available in our rural location. Climate data and cloud coverage for each location on the day of sampling as well as the monthly average temperatures and precipitation was obtained from https://www.timeanddate.com/.

### DNA extraction and sequencing

Dissected gut tissue was removed from RNAlater, washed with ethanol, and dried at room temperature for 5 min. Samples were then homogenized and a phenol chloroform DNA extraction with bead beating was performed for each individual bee gut as in [14]. Of the 144 samples collected, we were able to successfully extract DNA from a total of 101 samples including one negative control. For sample information see Additional file 4: Dataset S3. Extracted DNA was used to perform a 2-step 16S rDNA library preparation [50]. For the 1st-step, PCR amplification of the V4 region of the 16S rRNA gene was done using the primers 515F and 806R with illumina platform specific sequence adaptors attached: Hyb515F_rRNA: 5'-TCGTCGGCAGCGTCAGATGTGTATAAGAGACAG**GTGYCAGCMGCCGCGGTA** -3' and Hyb806R_rRNA: 5'-GTCTCGTGGGCTCGGAGATGTGTATAAGAGACAG**GGACTACHVGGGTWTCTAAT**-3'. PCR cycling conditions were 98 °C for 30 s followed by 20 cycles of 98 °C (10 s), 58 °C (30 s), 72 °C (30 s), with a final extension at 72 °C for 7 m. The resulting PCR product was cleaned using a Axygen™ AxyPrep Mag™ PCR Clean-up Kit. For the 2nd step, the amplicons were indexed using the Illumina Nextera XT Index kit v2 set A. PCR cycling conditions were 98 °C for 2 m followed by 15 cycles of 98 °C (10 s), 55 °C (30 s), 72 °C (30 s), with a final extension at 72 °C for 7 m. The final indexed amplicons were cleaned using the a Axygen™ AxyPrep Mag™ PCR Clean-up Kit, quantified with a Qubit3.0 (Life Technologies) with the Qubit dsDNA BR Assay kit, and pooled in equal concentrations for sequencing. A PhiX spike-in of 30% was added to the pooled library before sequencing to increase diversity on the run. Amplicon sequencing was performed in house using an Illumina iSeq100 with 2 × 150 paired end reads.

### Sequence analysis

The 16S amplicon sequencing was performed on the Illumina iSeq100. The total number of reads passing filter obtained from the sequencing run was 11,116,146 (approximately 5,500,000 forward and reverse reads each). Forward and reverse reads were merged using FLASH [51] with minimum overlap of 5 bp. Joined reads were quality filtered in Qiime2 [52] using the DADA2 [53] pipeline, which includes removal of PhiX and chimeric reads. The data was then filtered to remove all sequences corresponding to mitochondria, chloroplast, and unassigned taxa. Further filtering was performed to remove any taxa that were represented by fewer than 10 reads. After quality filtering, we obtained 1,556,467 reads with a mean frequency of 15, 400 reads per sample and 779 ASVs. The negative control contained 142 reads that represented five ASVs, consisting of only 0.009% of the total reads. Of the five ASVs found in the negative control, three were not found in any other sample and two were members of the core honey bee microbiome. To account for the contamination, the three unique ASVs were removed. Since the other two ASVs were core and present in high abundance in our samples, we subtracted the number of reads found in the negative control from the number of reads found in each sample. We used this modified read count for all downstream analyses.

Downstream analyses were performed in Qiime2 [52] at a sampling depth of 9000 reads per sample. This sampling depth was chosen to maximize the number of samples included in the analysis while still maintaining enough reads per sample to capture the richness of the dataset. Rarefying to 9000 reads per sample resulted in a total of 100 samples (35 rural RJBs, 34 urban RJBs, and 31 urban ITBs). A tree was then generated for phylogenetic diversity analysis using the script “qiime phylogeny align-to-tree-mafft-fasttree” [54, 55]. Alpha and beta diversity analyses were then conducted using the script “qiime diversity core-metrics-phylogenetic” [52]. Alpha and beta diversity group significance was tested using the scripts “qiime diversity alpha-group-significance” and “qiime diversity beta-group-significance” [52]. Beta diversity was analyzed using two different methods, Bray–Curtis dissimilarity and weighted unifrac. Bray–Curtis dissimilarity is a quantitative non-phylogenetic method for comparing differences between groups, whereas weighted unifrac is a quantitative metric that incorporates phylogenetic distances between observed organisms. Taxonomy was assigned to the representative sequences with the q2-feature-classifier plugin [56] using the curated database for bumble and honeybee gut microbiota, the Bee Gut Microbiota-Database (BGM-Db) [25]. The taxonomic assignment of poorly classified ASVs were manually verified using NCBI blastn [57]. Taxonomic diversity was analyzed at the genus and ASV level. For details on individual sample information, including relative abundance of taxa see Additional file 2: Datasets S1, Additional file 4: Datasets S2, Additional file 5: Datasets S3.

### Statistical analysis and data visualization

Statistical analyses of alpha diversity were conducted in Qiime2 (ref) using the Kruskal–Wallis test. Alpha diversity results generated in Qiime2 (ref) were plotted in R [58]. Statistical analyses of beta diversity were conducted in Qiime2 using the PERMANOVA test with 999 permutations. PCoA plots of beta diversity with 95% confidence intervals (stat_ellipse) were generated in using Qiime2R [59]. Differential abundance of taxa was tested using ANCOM [26] implemented in Qiime2. Pseudocounts were added to the data using “qiime composition add-pseudocount” before running ANCOM to remove zeros. ANCOM differential abundance volcano plots were generated in R [58]. All aesthetic modifications were performed in Adobe Illustrator.

## Supplementary Information

**Additional file 1: Figure S1:** Beta diversity comparisons of the gut microbiomes of urban and rural bees. Principal coordinate analysis and pairwise distance boxplots based on Bray Curtis dissimilarity and weighted Unifrac. Significance was tested using PERMANOVA with 999 permutations: ITBs versus RJBs (Bray Curtis, p = 0.083; weighted UniFrac, p = 0.19), rural RJBs versus urban ITBs (Bray Curtis, p = 0.016; weighted UniFrac, p = 0.037), rural RJBs versus urban RJBs (Bray Curtis, p = 0.167; weighted UniFrac, p = 0.016).

**Additional file 2: Dataset S1:** Relative abundance of each ASV present in sampled honey bee guts.

**Additional file 3: Figures S2:** ANCOM differential abundance volcano plots. The y-axis represents the W value or the number of times of the null-hypothesis was rejected for a given taxonomic cluster or ASV. The x-axis value represents the clr transformed mean difference in abundance of a given taxon or ASV. No Taxa/ASVs were found to be significant. Taxa/ASVs that were not significant but displayed a higher than average W value are labeled with taxon name.ANCOM differential abundance volcano plots. The y-axis represents the W value or the number of times of the null-hypothesis was rejected for a given taxonomic cluster or ASV. The x-axis value represents the clr transformed mean difference in abundance of a given taxon or ASV. No Taxa/ASVs were found to be significant. Taxa/ASVs that were not significant but displayed a higher than average W value are labeled with taxon name.

**Additional file 4: Dataset S2:** Dataset S2: Relativeabundance of ASVs present in sampled honey bee guts clustered by taxonomy.

**Additional file 5: Dataset S3:** Metadata table for samples used in this study

## Data Availability

16S rRNA gene amplicon reads were deposited in the Sequence Read Archive (SRA) at NCBI under PRJNA702133. All other data generated or analyzed during this study are included in this published article [and its supplementary information files].
