# Supplementary figures and images for: High royal jelly production does not impact the gut microbiome of honey bees
Source: Anim Microbiome. 2021 Sep 13;3:60. doi: 10.1186/s42523-021-00124-1 (PMC8439078; doi:10.1186/s42523-021-00124-1)

**Bray Curtis Dissimilarity**

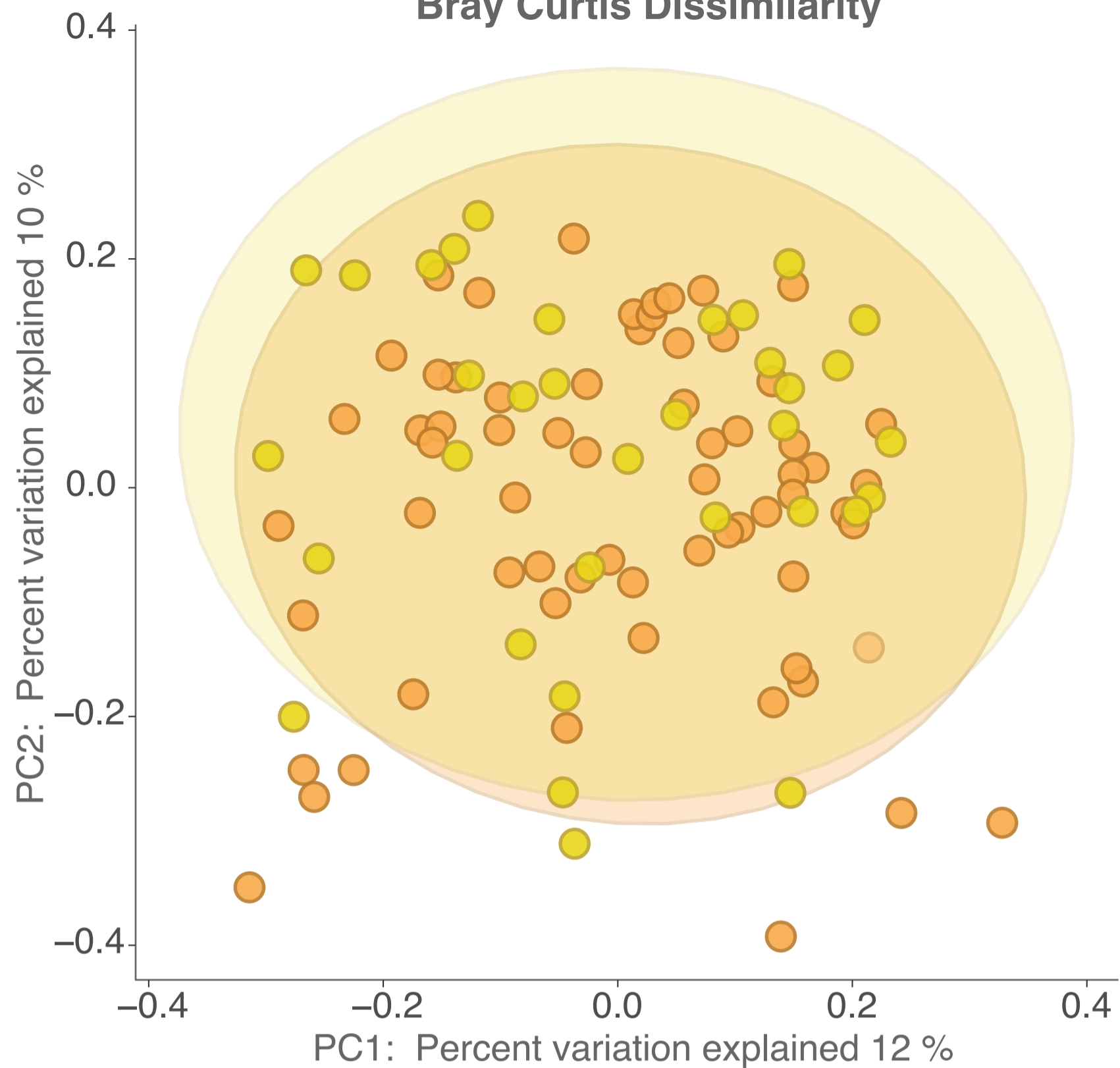

**Bray Curtis Dissimilarity**

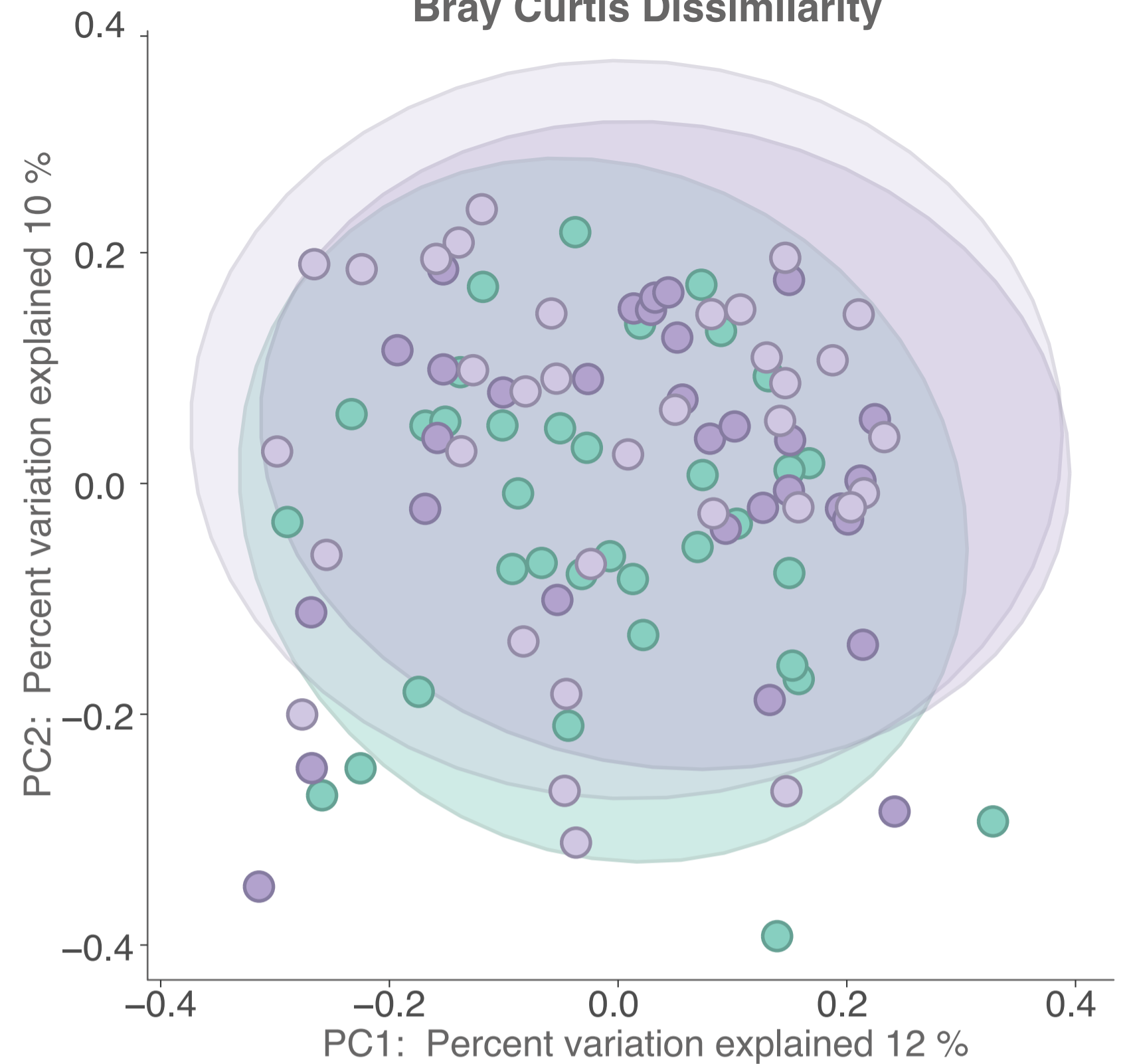

**Weighted UniFrac**

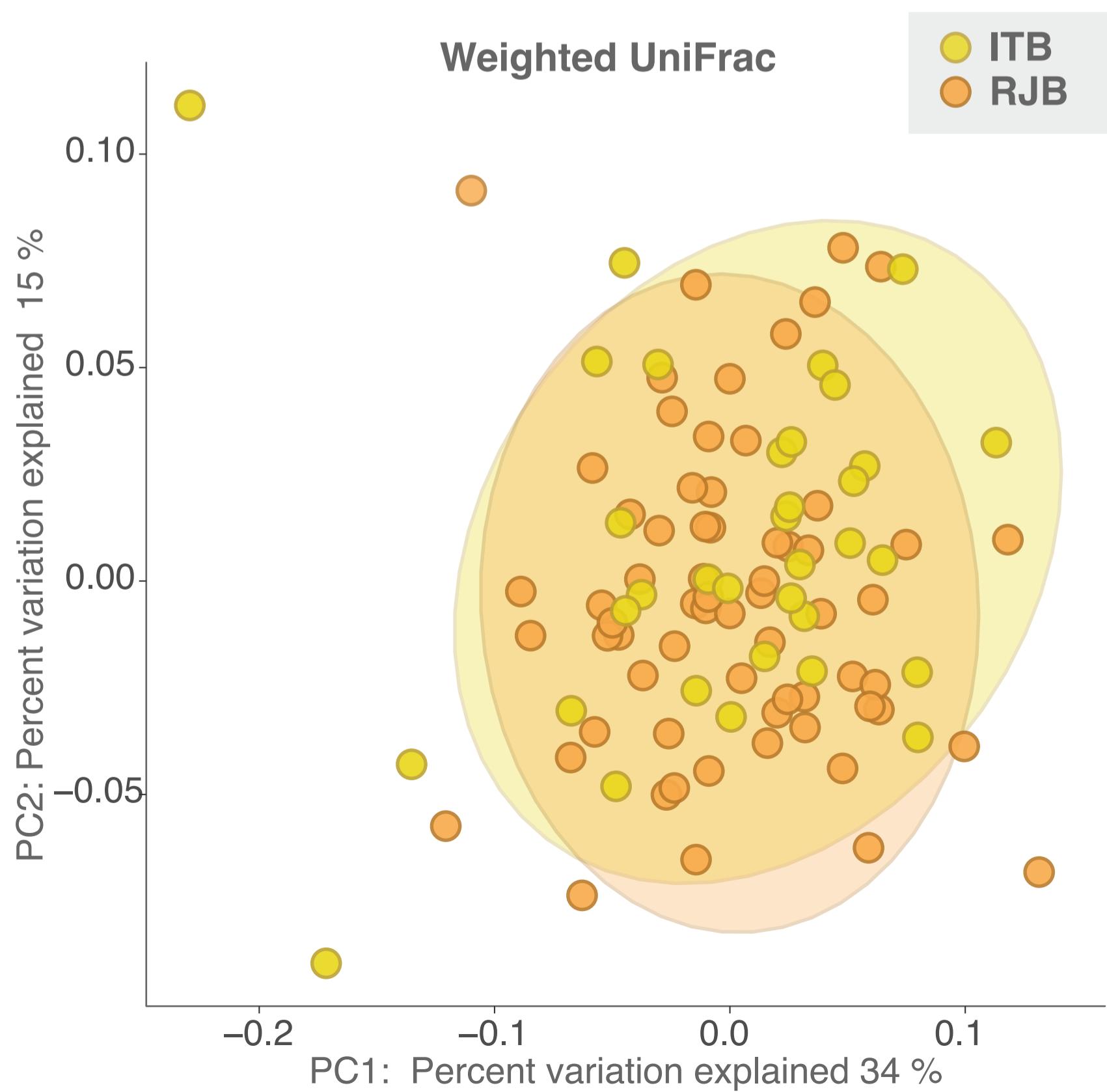

**Weighted UniFrac**

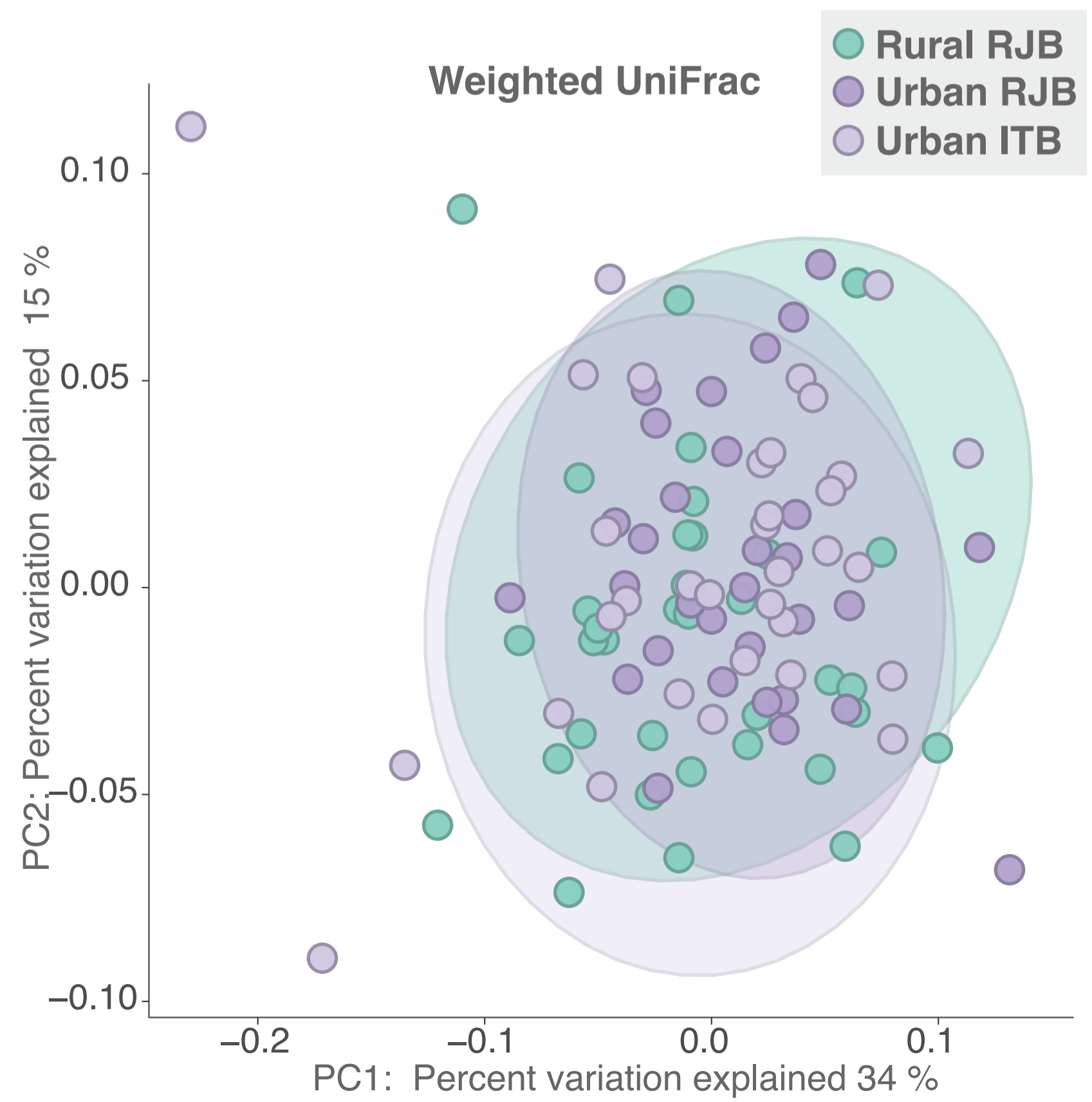

Supplement: Supplementary file 1 — Additional file 1: Figure S1: Beta diversity comparisons of the gut microbiomes of urban and rural bees. Principal coordinate analysis and pairwise distance boxplots based on Bray Curtis dissimilarity and weighted Unifrac. Significance was tested using PERMANOVA with 999 permutations: ITBs versus RJBs (Bray Curtis, p = 0.083; weighted UniFrac, p = 0.19), rural RJBs versus urban ITBs (Bray Curtis, p = 0.016; weighted UniFrac, p = 0.037), rural RJBs versus urban RJBs (Bray Curtis, p = 0.167; weighted UniFrac, p = 0.016). [file 42523_2021_124_MOESM1_ESM.pdf]

Urban RJB vs Urban ITB

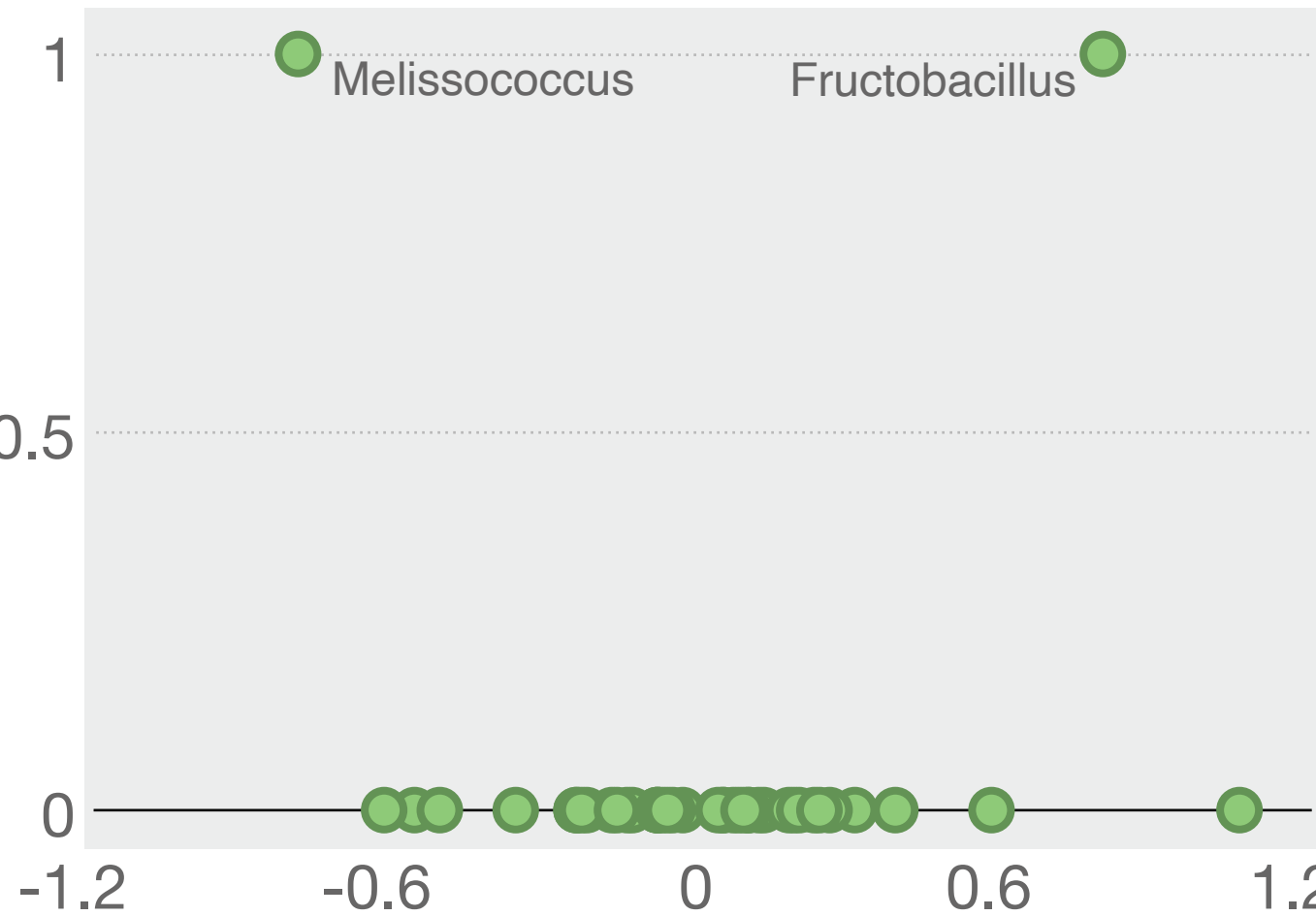

Urban RJB vs Urban ITB

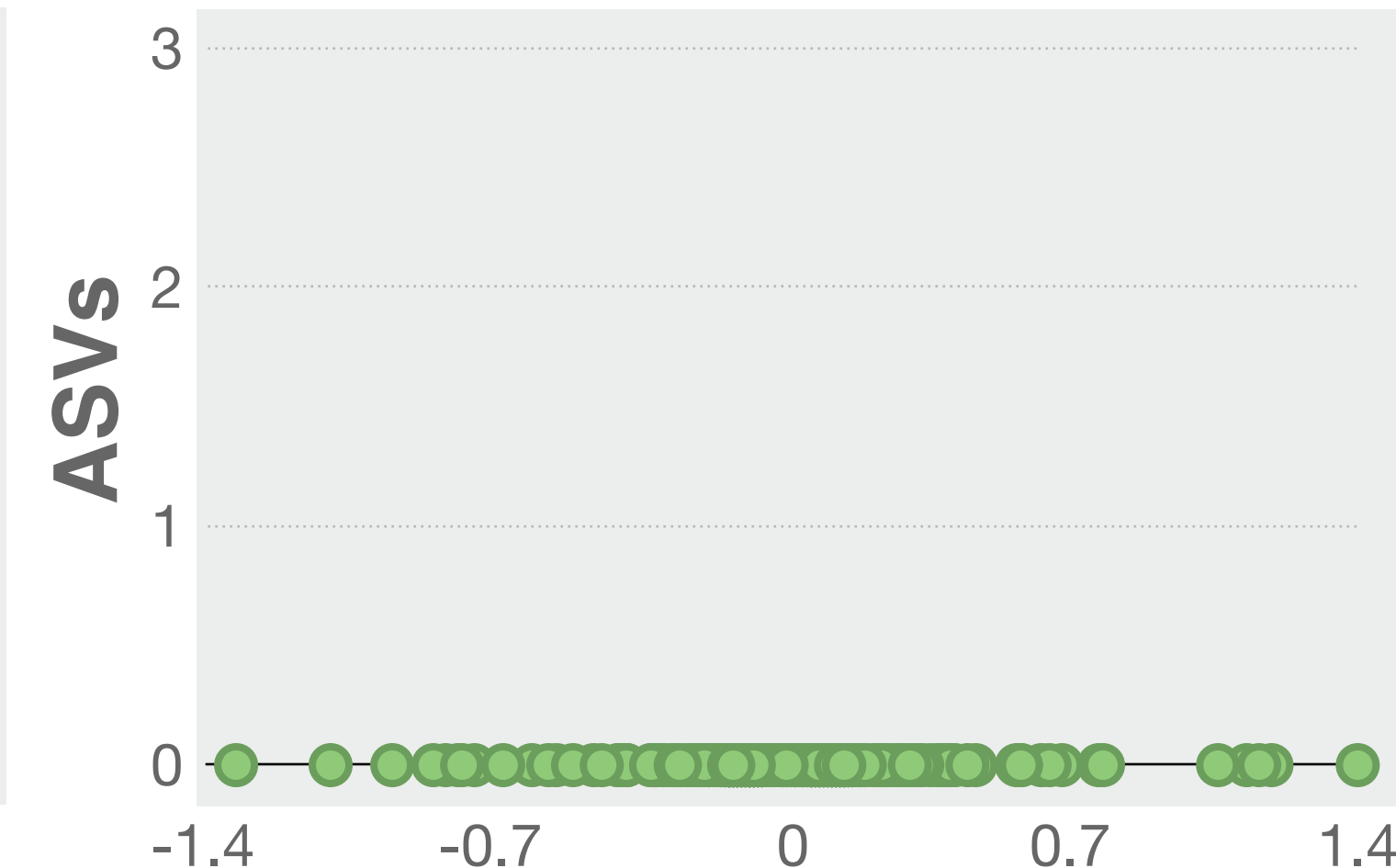

Urban RJB vs Rural RJB

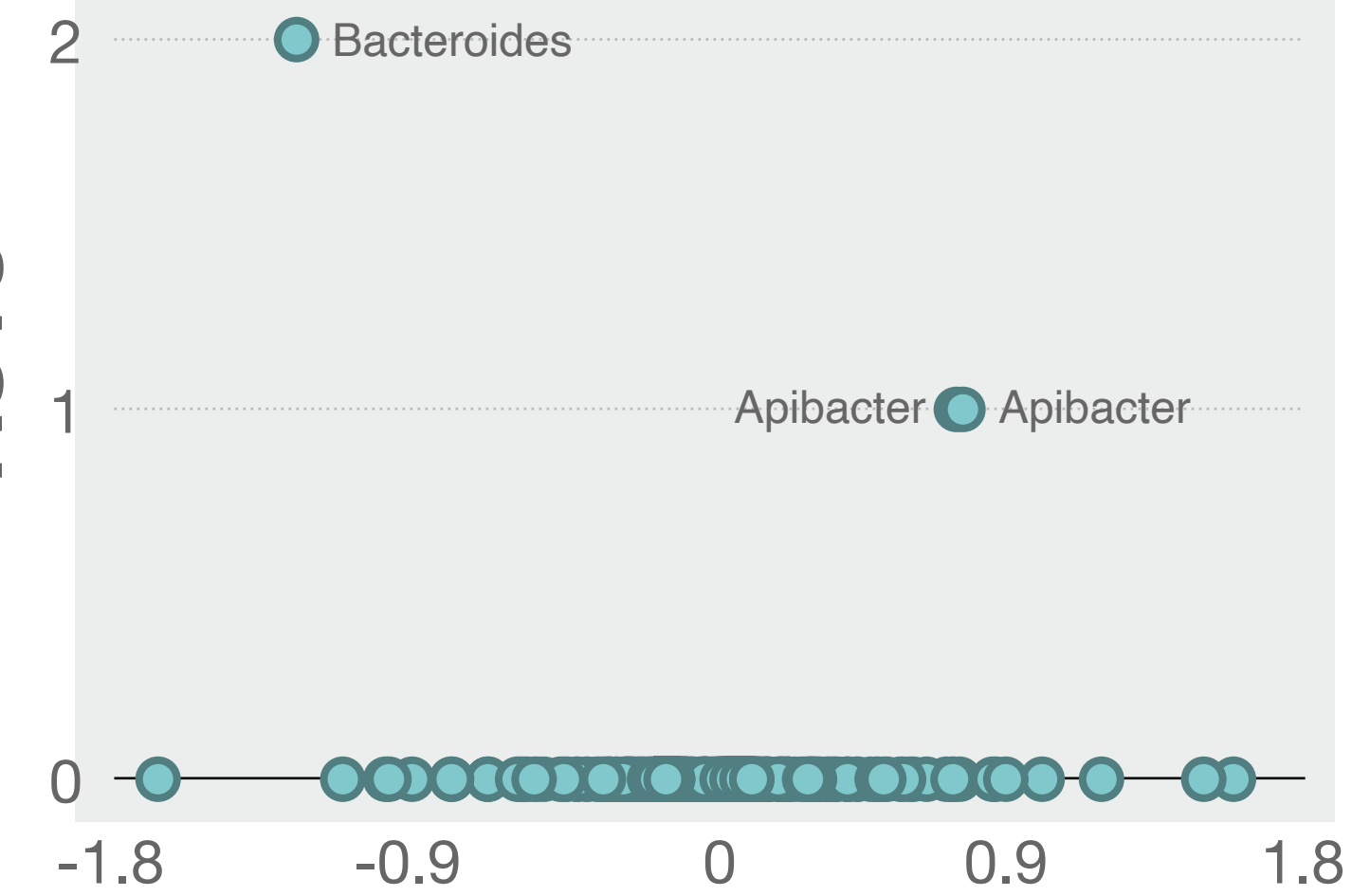

RJB vs ITB

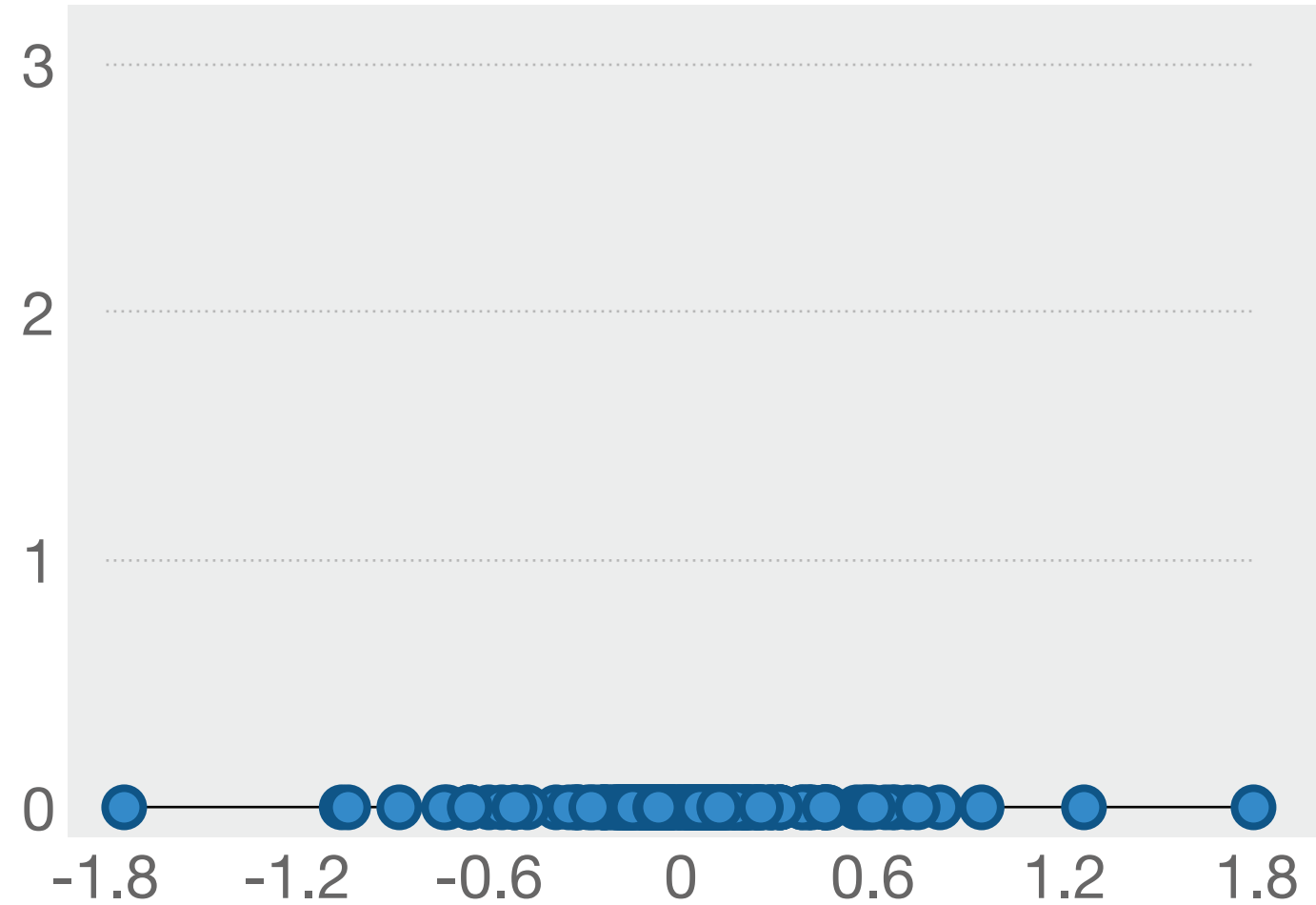

Supplement: Supplementary file 3 — Additional file 3: Figures S2: ANCOM differential abundance volcano plots. The y-axis represents the W value or the number of times of the null-hypothesis was rejected for a given taxonomic cluster or ASV. The x-axis value represents the clr transformed mean difference in abundance of a given taxon or ASV. No Taxa/ASVs were found to be significant. Taxa/ASVs that were not significant but displayed a higher than average W value are labeled with taxon name.ANCOM differential abundance volcano plots. The y-axis represents the W value or the number of times of the null-hypothesis was rejected for a given taxonomic cluster or ASV. The x-axis value represents the clr transformed mean difference in abundance of a given taxon or ASV. No Taxa/ASVs were found to be significant. Taxa/ASVs that were not significant but displayed a higher than average W value are labeled with taxon name. [file 42523_2021_124_MOESM3_ESM.pdf]
